# Satisfaction among edentulous patients before and after conventional complete denture

**DOI:** 10.6026/973206300210463

**Published:** 2025-03-31

**Authors:** Rahul A Razdan, Rajeev Srivastava, Trapti Jaiswal, Monica Garg, Harsh Chansoria, Krati Bais

**Affiliations:** 1 Department of Prosthodontics and Crown & Bridge, Index Institute of Dental Sciences, Indore, Madhya Pradesh, India; 2 Department of Prosthodontics and Crown & Bridge, Sri Aurobindo College of Dentistry, Indore, Madhya Pradesh, India; 3 Department of Prosthodontics and Crown & Bridge, Government College of Dentistry, Indore, Madhya Pradesh, India

**Keywords:** Edentulism, complete dentures, patient satisfaction, smile confidence, eating comfort

## Abstract

Satisfaction levels in edentulous patients before and after receiving conventional complete dentures, focusing on smile confidence, eating comfort and pronunciation is of interest. Hence, a cross-sectional analysis of 100 patients (aged 52-75 years) using a structured questionnaire was completed to assess post-treatment improvements. Confidence in smiling increased from 8% to 62%, while eating comfort and pronunciation improved in 67% and 58% of patients, respectively. Thus, transformative impact of complete dentures on both functionality and psychosocial well-being is highlighted. Therefore, comprehensive care and patient education are essential to optimizing treatment outcomes.

## Background:

Edentulism, the complete loss of natural teeth, is a widespread condition that profoundly affects an individual's physical, social and emotional well-being [1]. The condition often leads to compromised mastication, speech difficulties and a loss of facial esthetics, which can significantly reduce the overall quality of life [2]. Additionally, edentulism may impact self-esteem and social interactions, as individuals may feel less confident in their appearance or ability to communicate effectively [3]. These challenges emphasize the critical need for prosthetic solutions that not only restore oral functionality but also address the psychological and social implications of tooth loss [4]. Complete dentures have long been the primary treatment modality for managing edentulism [5]. They aim to restore essential oral functions, including chewing and speech, while improving the esthetic appearance of the oral cavity [6]. However, achieving optimal patient satisfaction with complete dentures can be challenging due to the inherent limitations of the prostheses and variations in individual adaptation [7]. Factors influencing satisfaction include the fit and comfort of the dentures, the patient's ability to adapt to the new prosthesis and the restoration of normal oral functions. The adaptability of the mucosal tissues, psychological acceptance and pre-existing oral health conditions also play a pivotal role in determining the effectiveness of the dentures [8].

Patient satisfaction serves as a comprehensive indicator of treatment success, encompassing functional, esthetic and psychological outcomes [9]. Functionally, the ability to chew effectively and speak clearly is critical for patients transitioning from an edentulous state. Esthetically, a restored smile can improve facial appearance and positively influence social interactions. Psychologically, the boost in self-esteem from regaining oral functions and esthetics can enhance the overall quality of life [10]. Measuring patient satisfaction before and after complete denture placement is essential for dental practitioners to evaluate the efficacy of their treatment approaches. It also provides critical insights into areas that require improvement, such as denture design, material advancements and patient education. Furthermore, understanding patient experiences can inform innovations in denture fabrication techniques, including digital workflows and advanced materials, to enhance patient outcomes [11]. Therefore, it is of interest to assess and compare the confidence levels and functional experiences of edentulous patients before and after receiving conventional complete dentures, with a focus on three domains: smile confidence, eating comfort and pronunciation difficulties.

## Methodology:

This cross-sectional study was conducted on 100 edentulous patients aged 52-75 years to evaluate and compare their satisfaction levels before and after receiving conventional complete dentures. The sample included a sex ratio of 58 females to 42 males. A structured questionnaire comprising nine items was used to assess three domains: confidence in smile, ability to eat and pronunciation difficulties, both before and after denture placement. Responses were recorded using a four-point Likert scale, ranging from "Very Confident" to "Not Confident at All" or from "Rarely or Never" to "Always," depending on the question. Data collection occurred over a period of three months in a clinical setting. Ethical approval was obtained and written informed consent was secured from all participants. Statistical analysis was performed using descriptive methods to identify trends and changes in satisfaction levels. Results were presented in tabular and heat map formats for clear visualization of the data. Data collection involved administering the questionnaire to participants during their clinical visits, with responses recorded immediately to ensure accuracy and reliability (Table 1 and Table 2).

## Results:

(Figure 1 - see PDF) The study revealed significant improvements in patient satisfaction across all evaluated domains after receiving conventional complete dentures. Confidence in smile showed a remarkable increase, with 62% of participants reporting feeling "very confident" post-denture, compared to only 8% before. Conversely, the proportion of those who felt "not confident at all" dropped sharply from 65% to just 7%. Similarly, confidence in eating comfortably demonstrated substantial growth; 51% of participants felt "very confident" post-denture, a significant rise from only 5% before the intervention. The percentage of individuals "not confident at all" about eating decreased dramatically from 70% to 5%. In terms of difficulties with pronunciation, improvements were also notable. The proportion of participants who rarely or never experienced difficulties increased from 15% to 55% after receiving dentures, while those who always faced pronunciation challenges dropped significantly from 51% to 14%. When comparing the pre-denture and post-denture conditions, most participants reported significant improvement in their overall satisfaction, particularly in confidence related to smile and eating, as well as reduced pronunciation challenges. These results emphasize the effectiveness of conventional complete dentures in enhancing the quality of life and restoring functionality for edentulous patients.

## Discussion:

The use of conventional complete dentures plays a pivotal role in improving oral functionality and enhancing psychosocial well-being for individuals with edentulism [12]. McGrath and Bedi demonstrated that these dentures significantly enhance the quality of life, both physically and psychologically, for denture wearers [13]. A study found that 60% of experienced denture wearers were able to function satisfactorily within a week after receiving new dentures, while an additional 20% required up to a month to fully adapt [7]. These findings emphasize the short-term adaptation period required for denture wearers to regain functionality and comfort. Moreover, Seenivasan *et al.* found that patients were generally satisfied with the quality of their complete dentures, with only a small percentage (7.2%) expressing absolute dissatisfaction, underscoring the overall positive impact of dentures on the patient experience [14]. Factors such as gender and previous denture experience have also been found to influence patient satisfaction, particularly in terms of psychological and social well-being [15]. A study noted that individuals with prior denture experience and those who received personalized care had higher satisfaction levels, particularly regarding emotional and social aspects of their lives. This demonstrates the importance of individualized care and patient education in maximizing the benefits of complete dentures [15, 16].

The study's findings align with existing literature, which consistently highlights significant improvements in patient satisfaction following the use of conventional complete dentures. For instance, a study published by Oweis *et al.* who reported that 60% of experienced denture wearers were able to function satisfactorily within a week after receiving new dentures, with an additional 20% requiring up to a month to adapt fully [7]. Celebic *et al.* who stated that, patients were generally satisfied with the quality of their complete dentures, with only 7.2% expressing absolute dissatisfaction [16]. Soboleva *et al.* concluded that patient satisfaction with complete dentures is primarily influenced by comfort and aesthetics, rather than age, sex, or degree of bone resorption. Stability was a key factor in mandibular denture comfort, which in turn affected overall satisfaction. Differences in satisfaction were noted between maxillary and mandibular dentures, particularly regarding chewing efficiency and stability [17]. Furthermore, a study by Oweis *et al.* in which they indicated that factors such as gender and previous denture experience significantly influenced patient satisfaction, particularly concerning psychological and social aspects [7]. These studies underscore the transformative impact of complete dentures on oral functionality and psychosocial well-being, highlighting the importance of personalized care and patient education to optimize treatment outcomes [7, 16].

## Conclusion:

Conventional complete dentures significantly improve both oral functionality and psychosocial well-being for edentulous patients. Most individuals experience high levels of satisfaction, with improvements in daily functioning and quality of life. Factors such as prior denture experience and personalized care play a key role in enhancing patient satisfaction, particularly in psychological and social aspects. These findings highlight the importance of individualized care and patient education to optimize the benefits of complete dentures, ultimately improving the overall quality of life for denture wearers.

## Figures and Tables

**Table 1:** Questionnaire design and distribution of questions

**Domain**	**Question**	**Response Options**
Confidence in Smile	Before receiving complete dentures, how confident do you feel about your smile?	A. Very Confident B. Somewhat Confident C. Neutral D. Not Confident at All
	After receiving complete dentures, how confident do you feel about your smile?	A. Very Confident B. Somewhat Confident C. Neutral D. Not Confident at All
	Comparing your smile confidence before and after dentures, how would you rate the change?	A. Improved Significantly B. Improved Somewhat C. No Significant Change D. Declined
Confidence in Eating Comfort	Before getting complete dentures, how confident are you in your ability to eat comfortably?	A. Very Confident B. Somewhat Confident C. Neutral D. Not Confident at All
	After receiving complete dentures, how confident are you in your ability to eat comfortably?	A. Very Confident B. Somewhat Confident C. Neutral D. Not Confident at All
	Comparing you is eating confidence before and after dentures, how would you rate the change?	A. Improved Significantly B. Improved Somewhat C. No Significant Change D. Declined
Pronunciation Difficulties	Before having complete dentures, do you experience difficulties in pronunciation?	A. Rarely or Never B. Occasionally C. Frequently D. Always
	After getting complete dentures, do you still experience difficulties in pronunciation?	A. Rarely or Never B. Occasionally C. Frequently D. Always
	Comparing your pronunciation difficulties before and after dentures, how would you rate the change?	A. Improved Significantly B. Improved Somewhat C. No Significant Change D. Declined

**Table 2:** Steps in methodology

**Step**	**Description**
Sample Size and Inclusion	The study included 100 edentulous patients aged 52-75 years, with a sex ratio of F:M = 58:42.
Data Collection Tool	A structured questionnaire with nine items focused on smile confidence, eating comfort and pronunciation.
Response Recording	Responses were recorded on a four-point Likert scale tailored to the question type.
Ethical Considerations	Ethical clearance was obtained and written informed consent was secured from all participants.
Data Analysis	Descriptive statistical methods were applied to compare responses before and after the placement of dentures.
Presentation of Data	Results were visualized using tables and heat maps to highlight the changes across satisfaction domains.
This structured approach ensured a
thorough evaluation of patient satisfaction before and
after receiving conventional complete dentures.
Annexure - questionnaire
